# Cryptobrachytone C from *Cryptocarya pulchrinervia* (Kosterm) Leaves on Proliferation, Apoptosis, Migration and Clonogenicity of MCF-7 and T47D Cell Lines

**DOI:** 10.21315/tlsr2023.34.2.11

**Published:** 2023-07-21

**Authors:** Jujun Ratnasari, Marselina Irasonia Tan, Rizkita Rachmi Esyanti, Lia Dewi Juliawaty

**Affiliations:** 1 Biology Education Department, Universitas Muhammadiyah Sukabumi, Jl. R. Syamsudin SH No 50, Sukabumi 43113, Indonesia; 2 Animal Physiology and Developmental Biology and Biomedical Sciences, School of Life Sciences and Technology, Institut Teknologi Bandung, Jl. Ganeca No 10 Bandung 40116, Indonesia; 3 Plant Sciences and Biotechnology, School of Life Sciences and Technology, Institut Teknologi Bandung, Jl. Ganeca No 10 Bandung 40116, Indonesia; 4 Organic Chemistry, Chemistry Study Program, Institut Teknologi Bandung, Jl. Ganeca No 10, Bandung 40116, Indonesia

**Keywords:** *Cryptocarya pulchrinervia*, Proliferation, Apoptosis, Migration, MCF-7, T47D, Cryptobrachytone C

## Abstract

*Cryptocarya pulchrinervia* is an Indonesian indigenous plant that grows in Sumatra, Kalimantan and Papua. One of the new compounds extracted from this plant was cryptobrachytone C, which was known to be cytotoxic against cancer cells of Murine leukemia P388 with IC_50_ 10.52 μM. In this study, the cytotoxicity and anticancer properties of cryptobrachytone C on proliferation, apoptosis, migration and clone formation of MCF-7 and T47D breast cancer cell lines were examined, which had not previously been done before. The cytotoxicity of the compound was measured using an MTT (3- (4,5-dimethylthiazol-2- yl) -2,5-di-phenyl-tetrazolium bromide) assay. The cell proliferation was measured using a BrdU assay, and the cell apoptosis was measured using annexin-V FITC, while the cell migration was measured using a transwell filter. The cytotoxic test result demonstrated that cryptobrachytone C was cytotoxic against MCF-7 cells with IC_50_ 12.94 ± 0.32 μM but not against T47D cells with IC_50_ 65.33 ± 2.33 μM nor against normal MRC-5 cells with IC_50_ 122.57 ± 19.84 μM. The cell proliferation assay showed that cryptobrachytone C at IC_50_ concentration had antiproliferative properties against MCF-7 cancer cell lines (*p* < 0.05) but did not significantly reduce T47D cell proliferation (*p* < 0.07). Although the results of the cell apoptosis test showed that cryptobrachytone C could induce the apoptosis of the MCF-7 and T47D cells, it was insignificant (*p* > 0.05). The cell migration test showed that cryptobrachytone at IC_50_ concentrations could inhibit the migration of the MCF-7 and T47D cells. The clonogenic test showed that cryptobrachytone C at IC_50_ concentration can induce the inhibition of the formation of MCF-7 and T47D cell colonies. The cryptobrachytone C anti-cancer character was more signi icant on the MCF-7 cell line compared to the T47D. This study showed that cryptobrachytone C was cytotoxic and had potential as an anti-cancer compound against MCF-7 and T47D breast cancer cell lines.

**Highlights**

- Cryptobrachytone C is a new compound isolated from the indigenous Indonesian plant *Cryptocarya pulchrinervia*.
- Cryptobrachytone C inhibited cell proliferation, migration and clonogenicity of the MCF-7 and T47D breast cancer cell lines.
- Cryptobrachytone C induced apoptosis in the MCF-7 and T47D cell lines.

## INTRODUCTION

Cancer is a frightening disease, it is the second cause of death after heart disease. More than 3.5 million people die from cancer each year (Amin *et al*. 2009; Kaur *et al*. 2011), and by 2018 over 9.5 million people across the world had died (IARC-WHO 2020). Various approaches are used to overcome this disease, including the use of natural compounds as anti-cancer drugs. Natural compounds have become one of the promising sources of medicine because they come from renewable resources that have wide diversity.

Some natural compounds that have been used for anti-cancer treatment include vincristine and vinblastine isolated from *Catharanthus roseus*, colchicine isolated from *Colchicum autumnal*, taxol and paclitaxel isolated from *Taxus brevifolia*. Some promising anti-cancer compounds are andrographoly isolated from *Andrographis paniculata*, morindron isolated from *Morinda citrifolia* (Raina *et al*. 2014), acetogenin isolated from *Annona muricata* and mangosteen isolated from *Garcinia mangostana* (Amin *et al*. 2009). Many higher plants have the potential to produce anti-cancer compounds, including plants from the Lauraceae family. One genus from the Lauraceae family is *Cryptocarya*, which was traditionally used as pain relief medication.

Indigenous to Indonesian, *Cryptocarya* is known by the local name Medang or Huru. Some *Cryptocarya* has bioactive compounds, including 2-hydroxy-atherosperminine, which was isolated from *C. nigra*, massoia lactone isolated from *C. massoia*, which had antiplasmodial activity and laurolitsine from *C. archboldiana* was cytotoxic. Some of the *Cryptocarya*, like *C. costata, C. crassinervia, C. densiflora, C. nigra, C. nitens, C. scortechinii, C. massoia, C. sumatrana, C. sumbawaensis, C. sulawesiana, C. ferrea, C. oblongifolia* and *C. pulchrinervia*, have bioactive compounds that have not been studied, also their activity as an anti-cancer have not been studied yet. Some natural compounds, which had been isolated from *Cryptocarya* and have cytotoxic activity, can be seen in Table 1.

One indigenous Indonesian *Cryptocary*a species is *Cryptocarya pulchrinervia*, which has produced a new compound named cryptobrachytone C. This compound belongs to a group of compounds called pyrones (Juliawaty *et al*. 2020). Some of the pyrone compounds had cytotoxic activity (Table 2), such as displaying antifungal, antibiotic, neurotoxic and phytotoxic activity. For an example, a 2-pyrone compound that had antiproliferative properties and induced DNA damage in A549 lung cancer cells (Calderon-Montano *et al*. 2013). Pyrone compounds also had anti-cancer properties against ovarian cancer cell lines A2780 and K562 (Fairlamb *et al*. 2004). Cryptocaryone isolated from *C. concinna* root could induce apoptosis in Ca9-22 and CAL27 oral cancer cell lines (Chang *et al*. 2016) (Table 2), and cryptobrachytone D isolated from *C. brachythyrsa* had antiproliferative properties in breast cancer line MDA-MB-231 (Fan *et al*. 2019). Cryptobrachytone C extracted from *Cryptocarya pulchrinervia* has cytotoxic against cancer cells of murine leukemia P388 with IC_50_ 10.52 μM. But has not been investigate yet for cell proliferation, apoptosis, migration and clonogenicity in the other cancer cell lines. This study aimed to analyse cryptobrachytone C activities, as one of the pyrone compounds, towards the proliferation, apoptosis, migration and formation of MCF- 7 and T47D breast cancer cell colonies.

MCF-7 cells are one o the first breast cancer cell lines isolated in 1970, belonging to the luminal A subtype, possessing estrogen (ER+) and progesterone (PR+) receptors. The hormones estrogen, progesterone and glucocorticoids play a role in the prolieration, differentiation and development o MCF-7 cancer cells. MCF-7 cells have both ERα and ERβ, but MCF-7 cells express more ER than Erβ (Comsa *et al*. 2015; Hegde *et al*. 2016). Meanwhile, T47D cells, like MCF-7 cells, are also breast cancer cell lines that are responsive to the hormone estrogen, progesterone and glucocorticoids (Hegde *et al*. 2016; Yu *et al*. 2017) and belong to the luminal A subtype (Dai *et al*. 2017; Tungsukruthai *et al*. 2018).

## MATERIALS AND METHODS

### Materials

*Cryptocarya pulchrinervia* leaves were obtained from the Bogor Botanical Gardens. Two kilograms of fresh leaves were dried and macerated for 24 h three times in acetone. The leaves macerate was then extracted using methanol water (1:1) to separate chlorophyll from the extract. The extract was fractionated by vacuum liquid chromatography using *n*-hexane-ethyl acetate as the eluent, and the fractions were evaporated. An examination for the presence or absence of cryptobrachytone C was carried out by thin-layer chromatography (TLC) with pure cryptobrachytone C as the standard, and *n*-hexane-ethyl acetate as the eluent. The extract was then fractionated again by column silica 7731 (Merck) gravity chromatography using *n*-hexane-ethyl acetate as the eluent. The fraction was then purified by crystallisation, and the structure was determined with 1D NMR (1H-NMR, 13C-NMR) and 2D NMR (HSQC and HMBC) with CDCl_3_ as the eluent (Juliawaty *et al*. 2020). From this purification, a pure compound in the form of bone-white crystals was obtained.

### Antioxidant Activity

The antioxidant activity index (AAI) was measured using the DPPH method (2,2-diphenyl-1-picrylhydrazyl) (Scherer & Godoy 2009). The calculation of the percentage of the inhibition was measured by the following equation (Scherer & Godoy 2009).


(1) $$\%\;\text{inhibition}=\frac{\text{absorbance of control }(\text{DPPH})-\text{absorbance of sample}}{\text{absorbance of control}}\times 100$$

(2) $$\text{AAI}=\frac{\text{concentration of control }(\text{ppm})}{{\text{IC}}_{50}}$$

### Culture of MCF-7 and T47D Cell Lines

MCF-7 was cultured in RPMI (Roswell Park Memorial Institute) 1640 medium (Gibco) with L-glutamine, 10% fetal bovine serum (FBS), 200 μL erythromycin-kanamycin and 30 units of insulin per 100 mL. The T47D cells were cultured in the same medium but without insulin. The culture was incubated at 37°C with 5% CO_2_, with medium replacement every two days. For the treatment, the cells were harvested using 0.025% EDTA and 0.25% trypsin (Gibco).

### Cytotoxic Activity

Cytotoxic activity was measured with the MTT assay (3- (4,5-dimethylthiazol- 2-yl) -2,5-diphenyl-tetrazolium bromide) (C_18_H_16_¬BrN_5_S). The cells (2 × 10^4^) were planted in 100 μL RPMI 1640 media on a 96 well plate then incubated for 24 h at 37°C with 5% CO_2_. The culture media were then aspirated, and the cells were rinsed three times using phosphate buffer saline (PBS). The total of 200 μL media were mixed with different concentrations of cryptobrachytone C based on multiple dilutions for a pure compound (0.69; 1.39; 2.78; 5.55; 11.11; 22.21; 44.42 and 88.85 μM) and were added to each well. For the negative control, 0.5% of *Dimethyl sulfoxide* (DMSO) was used, and 0.9 μM of doxorubicin was used as a positive control. The cultures were then incubated for 48 h. The media were then aspirated, and 100 μL of the media were mixed with 0.5 μg/mL MTT. Then, the cultures were incubated for another 5 h at 37°C. After incubation, the media were aspirated, and the DMSO was added to stop the reaction. The DMSO also functions as a formazan resolvent. The dissolved formazan was measured with a microplate reader at a wavelength of 595 nm. The cell viability was calculated using the following equation:

The control medium was the only medium (without cells), and the control solvent was the cell culture with the solvent (DMSO) added. The IC_50_ was analysed with the GraphPad Prism 8.02 programme.

### Bromodeoxyuridine (BrdU) Assay

The BrdU-ELISA assay (5-Bromo-2′-deoxyuridine) was used to determine cell proliferation based on the principle of labelling uridine incorporated into DNA during the S phase. The BrdU assay kit used was the Cell Proliferation Elisa BrdU (Colorimetric) Abcam ab126556 kit. As many as 2 × 10^4^ cells were plated in a 100 μL complete RPMI medium and incubated for 24 h. After 24 h, the cells were treated with cryptobrachytone C, for positive control cell treated with doxorubicin 0.9 μM, and cells culture without treatment as negative control. After treatment, 20 μL BrdU was added. The cells were harvested for 6 h, 12 h and 24 h after the BrdU treatment. The harvested cells were treated with a fixing solution for 30 min and then given a detector anti-BrdU monoclonal antibody and incubated at room temperature for 60 min. Next, the 100 μL of horseradish peroxidase-conjugated goat anti-mouse IgG was added and incubated for 30 min at room temperature under closed conditions. Then, 100 μL tetramethylbenzidine (TMB) was added and incubated at room temperature for 30 min in dark conditions. After 30 min, 100 μL of a stopping reagent was added, and the absorbance was measured with a spectrophotometer using dual wavelengths of 450/595 nm.

### Apoptosis Assay

The cells were plated onto a 6-well plate in the RPMI medium (Gibco) and incubated for 24 h. After incubation the cell then treated with a concentration series of cryptobrachytone C (IC_25_, IC_50_ and IC_75_), the concentrations series based on the results of the MTT assay. Some cell treated with doxorubicin 0.9 μM for positive control and untreated cells for negative control and incubated for 20 h. After 20 h of incubation, the cultures were treated with trypsin and were centrifuged. The cell pellets were resuspended in 1 mL medium then centrifuged again at 2.000 rpm for 5 min. The cell pellets were then resuspended with 300 μL PBS and transferred to a microtube and centrifuged at 2.000 rpm for 3 min. They were resuspended with a 100 μL binding buffer and then 5 μL fluorescein isothiocyanate (FITC) annexin, and 5 μL propidium iodide (PI) was added and then vortexed. The suspensions were then incubated at room temperature in the dark for 15 min after which a 400 μL binding buffer was added to each tube and transferred to the flow cytotube, and the fluorescence of the cells were measured on a flow cytometer (BD Accuri C6 +).

### Clonogenic Assay

Fifty MCF-7 cells and 100 T47D cells were planted in an RPMI medium (Gibco) per well in a 6-well plate then incubated for 24 h at 37°C and 5% CO_2_. After incubation, the cells were treated with cryptobrachytone C, treated with doxorubicin 0.9 μM as a positive control and given no treatment (cells only) as a negative control. The culture was then incubated for one to two weeks or until a colony was formed, and the cells were then fixed with 6% glutaraldehyde and stained with 0.5% crystal violet. The staining was done for 30 min after which the cells were washed with tap water and dried at room temperature. After drying, the colonies were observed under an inverted microscope. The calculations for the plating efficiency (PE) and the surviving fraction (SF) used the following equations (Franken *et al*. 2006).


$$\begin{array}{c} \text{PE}=\frac{\left(\text{Number of colonies formed}\right)}{\left(\text{Number of cells planted}\right)}\times 100\%\\ \text{SF}=\frac{\left(\text{Number of colonies formed after treatment}\right)}{\left(\text{Number of cells planted}\right)}\times 100\% \end{array}$$

### Cell Migration Assay

The cells were cultured in a serum-free medium and supplemented with 0.1% BSA (bovine serum albumin). As many as 2 × 10^5^ cells in a 100 μL medium were then planted onto the Transwell filters that had been inserted into a 24-well plate. The wells were filled with 600 μL complete media and incubated for 10 min at 37°C and 5% CO_2_. After incubation, the cultures were treated with cryptobrachytone C, treated with doxorubicin 0.9 μM as a positive control, given no treatment (cells only) as a negative control and then reincubated for 48 h at 37°C and 5% CO_2_. After incubation, the cells were fixed with 6% glutaraldehyde and stained with 0.5% crystal violet. The cells in the Transwell were observed under an inverted microscope, and cell colony counting was performed using ImageJ software.

### Data Analysis

Analysis data in the research were using software SPSS 25.

## RESULTS

Cryptobrachytone C was isolated from the *C. pulchrinervia* leaves. The form of the compound is white bone crystals. It had moderate antioxidant activity with an AAI of 0.57. This compound was cytotoxic to the MCF-7 breast cancer cell line with an IC_50_ concentration of 12.94 ± 0.32 μM. It was not toxic to the T47D breast cancer cell line with an IC_50_ concentration of 65.33 ± 2.33 μM and also the normal MRC-5 cells (Table 3).

**Figure 1:**
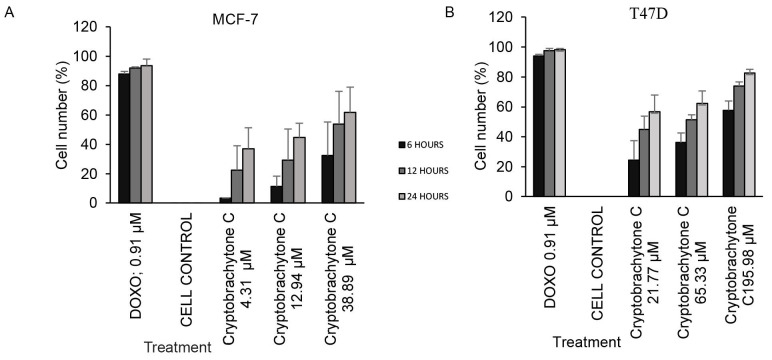
Cryptobrachytone C induced proliferation inhibition on MCF-7 at IC_50_ concentration (12.94 mM) 12 h after treatment (*p* < 0.05). The proliferated cell decreased as the cryptobrachytone C increased in concentration, even though not as significantly as the positive control doxorubicin 0.9 mM (A). Proliferation inhibition on T47D at IC_50_ concentration (65.33 mM) 12 h after treatment (B).

Cryptobrachytone C at a IC_50_ concentration (12.94 ± 0.32 μM) could inhibit MCF-7 cell proliferation. This could be seen in the decrease in the number of cells that proliferated compared to the negative control. A percentage reduction occurred at 12 h after treatment (Fig. 1A). Inhibition of the proliferation at 12 h after treatment at the IC_50_ of cryptobrachytone C was 29.33 ± 21.20%, compared to the positive control (doxorubicin 0.9 μM), which had a proliferation inhibition of 92.05 ± 0.69% (Table 4, Fig. 1A). T47D proliferation inhibition occurred 12 h after treatment with cryptobrachytone C at a IC_50_ concentration (65.33 ± 2.33 μM) (Fig. 1B). In the T47D cell line, the proliferation inhibition percentage is 51.42 ± 3.31% compared to the positive control (doxorubicin 0.9 μM) that inhibited 97.63 ± 1.40% (Table 5, Fig. 1B).

**Figure. 2:**
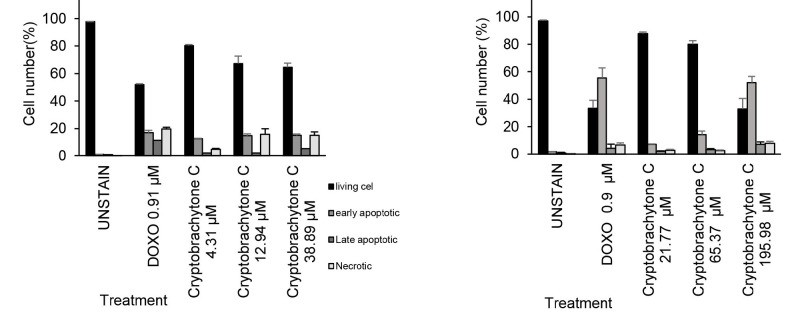
IC_50_ of cryptobrachytone C induced apoptosis and necrosis in MCF-7 and T47D. Percentage of living cell decreased in IC_50_ concentration but induced early and necrotic both in (A) MCF-7 and (B) T47D.

In addition to inhibiting MCF-7 and T47D cell proliferation, cryptobrachytone C influenced the induction of MCF-7 and T47D cell apoptosis, but the effect was not significant (*p* > 0.05) (Tables 6 and 7, Fig. 2). The difference in cytotoxicity of cryptobrachytone C in MCF-7 and T47D could be caused by differences in the characteristics of the two cell lines. Although both belong to the ER+ and PR+ subtypes, each has a different sensitivity to estrogen. MCF-7 cells do not respond to progesterone in the presence of estrogen, while T47D cells respond more positively to progesterone than to estrogen. Therefore, changes in the expression of the PR target gene STAT5A only occur in T47D and not in MCF-7 (Dai *et al*. 2017; Yu *et al*. 2017). Yu *et al*. (2017) have also reported that T47D cells are a more appropriate experimental model to elaborate the effect of progesterone on luminal A subtype breast cancer cells.

Cryptobrachytone C at a IC_50_ concentration induced apoptosis in MCF-7. Early apoptosis induced for 14.75 ± 1.39%, late apoptosis induced 2.17 ± 0.09% and necrosis induced 15.75 ± 4.08%, while the positive control (doxorubicin 0.9 μM) induced early apoptosis 16.91 ± 1,78 %, late apoptosis 11.35 ± 0.02% and necrosis 19.78 ± 1.14% (Table 6, Fig. 2A).

As for T47D, the IC_50_ concentrations of Cryptobrachytone C (65.32 μM) induced early apoptosis 14.17 ± 2.64%, late apoptosis 3.37± 0.66% and necrosis 2.42 ± 0.59% (Table 7, Fig. 2B), while the positive control (doxorubicin 0.9 μM) induced early apoptosis 55.58± 7.24%, late apoptosis 4.27 ± 3.02% and necrosis 6.67± 1.51% (Table 7, Fig. 2B).

**Figure 3:**
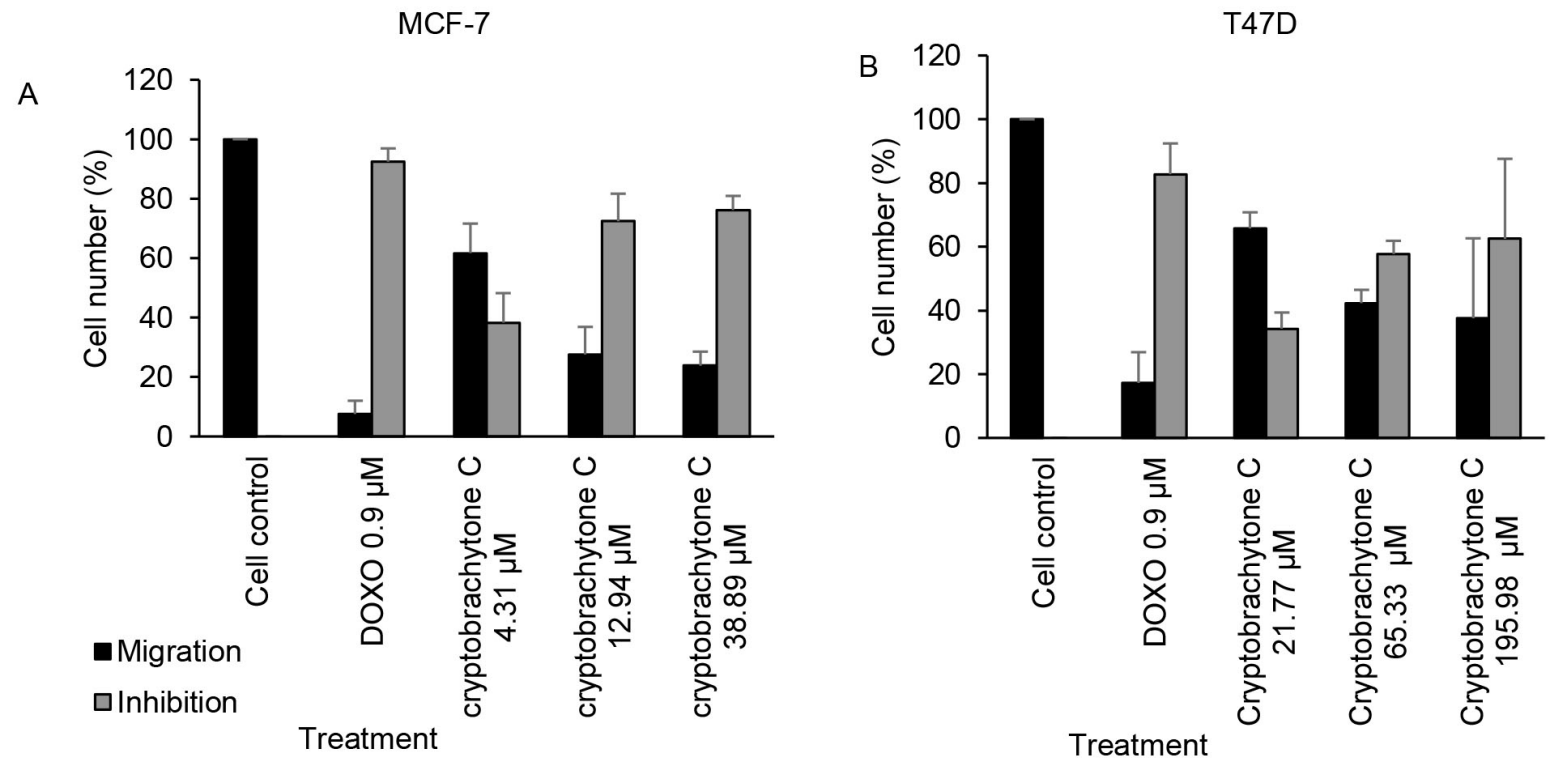
Cryptobrachytone C at IC_50_ (12.94 μM) inhibited cell migration significantly in (A) MCF-7 (*p* < 0.05), while in (B) T47D, inhibited cell migration at IC_50_ cryptobrachytone C (65.33 μM) is not significant (*p* = 0.07).

**Figure 4:**
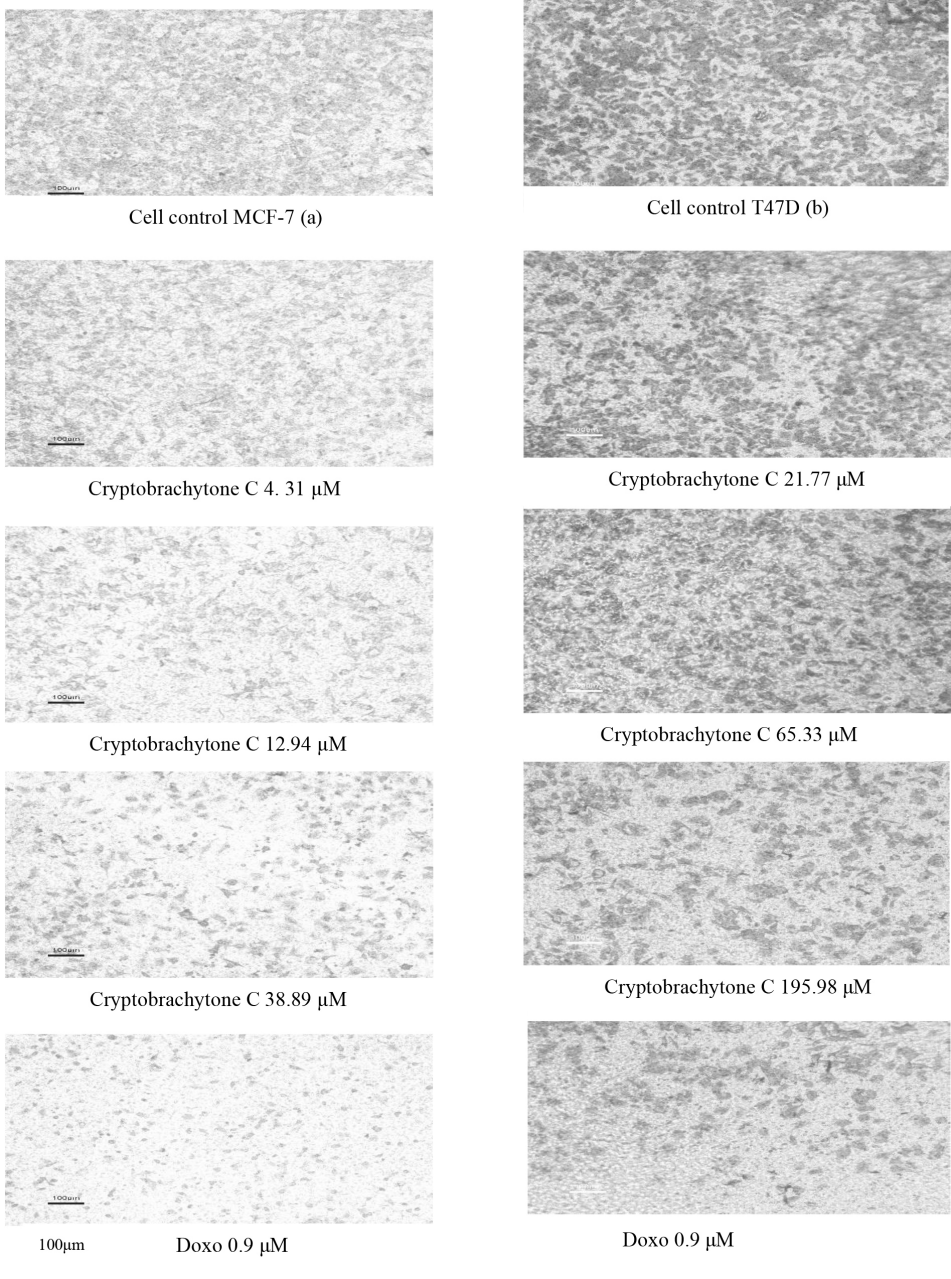
Migration inhibition in (A) MCF-7 and (B) T47D cell lines. Percentage cell migration decreased on a higher concentration of cryptobrachytone C both in MCF-7 and T47D (Obj. 10×).

**Figure 5:**
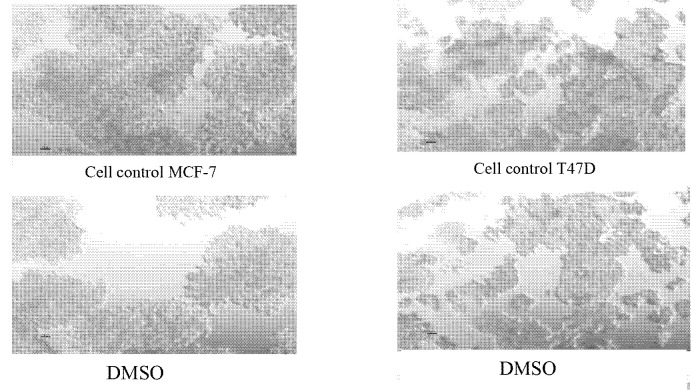
Colonies formation of (A) MCF-7 and (B) T47D cell lines. The number and size of the colonies decreased on a higher concentration of cryptobrachytone C both in MCF-7 and T47D (Obj. 40×).

**Figure 6:**
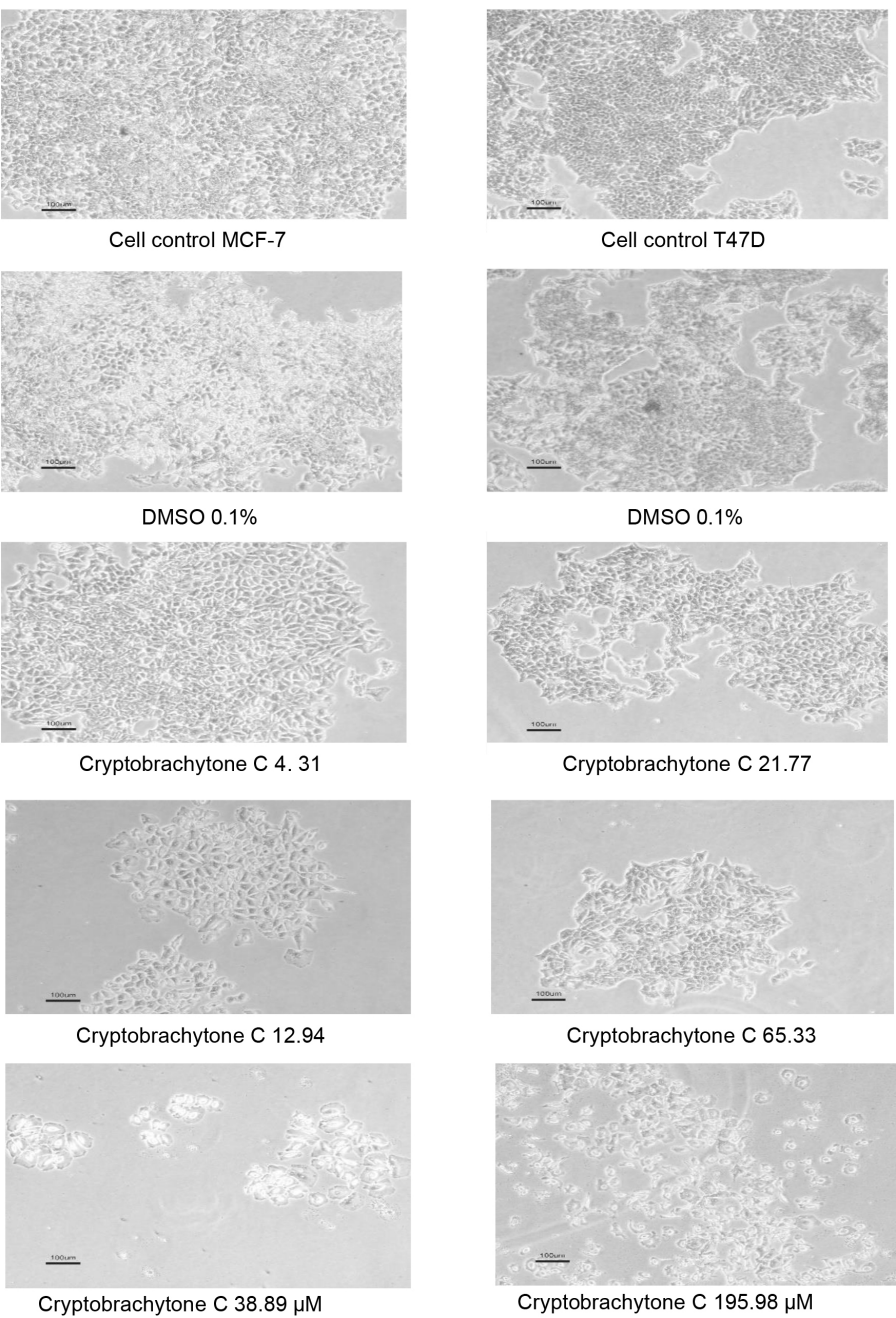
Colonies of (A) MCF-7 and (B) T47D showed decrease in density and number of the cell on a higher concentration of cryptobrachytone C both in MCF-7 and T47D (Obj. 100×).

The effect of IC_50_ c ryptobrachytone C could inhibit the c ell migration of MCF-7 and T47D. The percentage of the migration inhibition of MCF-7 is 72.41 ± 9.27%, while T47D is 57.79 ± 4.16 % (Table 8). The percentage of the cell migration in both cell lines decreased as the cryptobrachytone C concentration increased (Table 8, Figs. 3 and 4). The reduction of the cell migration compared significantly to a positive control (doxorubicin 0.9 μM). IC_50_ cryptobrachytone C could also inhibit colony formation in MCF-7 and T47D cell lines but not significantly. In MCF-7, the inhibition of colony formation began to be seen clearly at IC_50_ concentrations with a SF of 0.32 ± 0.04 compared with the SF of the control (DMSO 0.1%), that is 0.47 ± 0.04 (Table 9). The number and the size of the colonies formed decreased as the cryptobrachytone C concentration increased (Table 9, Figs. 5 and 6). As for the T47D cell line, the colonies were smaller than the MCF-7 colonies and began to form clearly at IC_50_ concentrations with SF 0.75 ± 0.16 compared with SF of the cell control as 0.76 ± 0.17 (Figs. 5 and 6).

## DISCUSSION

Cryptobrachytone C had moderate antioxidant activity, so the ability of cryptobrachytone C to oxidise free radicals was not very high. This could be caused by the cryptobrachytone C structure, which did not have many aromatic rings. The hydroxyl groups or the double bonds aromatic rings and hydroxyl groups has a better chance of binding free radicals. Therefore, if the chemical structure of a compound did not have many aromatic rings or hydroxyl groups, its ability to bind to free radicals would be reduced (Scherer & Godoy 2009). The compounds with many aromatic rings and double bonds had a greater chance of being substituted with the hydroxyl groups, and the hydroxyl groups had a greater chance of being substituted by the methoxyl groups. Moreover, the hydroxyl groups could more easily bind to hydroxyl, ferroxyl and feroxinitrile radicals (Hyun *et al*. 2010; Grigalius & Petrikaite 2017). In the research of Fan *et al*. (2019), cryptobrachytone C and cryptobrachytone D isolated from *Cryptocarya brachytyrsha* had similar chemical structures; the difference was only in the double bonds in C-11. It turned out that cryptobrachytone D was more cytotoxic against breast cancer MDA-MD-231 compared to cryptobrachytone C (Fan *et al*. 2019). This showed that in addition to the aromatic rings and hydroxyl groups, the number of double bonds in the compound also influenced the antioxidant activity of the compound. Therefore, for cryptobrachytone C, whose chemical structure only had a single aromatic ring, a single hydroxyl group and few double bonds, its antioxidant activity was only moderate.

Cryptobrachytone C isolated from *C. pulchrinervia* (an Indonesian indigenous plant) had cytotoxic properties against MCF-7 breast cancer cells with an IC_50_ concentration of 12.94 ± 0.32 μM, but it was less toxic to T47D with an IC_50_ concentration of 65.33 ± 2.33 μM and not toxic to normal cells MRC-5 (Table 3). Different cytotoxic effects of cryptobrachytone C could be caused by different target cell characteristics. MCF-7 and T47D are breast cancer cell line subtype IDC (invasive ductal carcinoma) luminal A with ER + PR + HER2- (Dai *et al*. 2017). This subtype is more sensitive to chemotherapy drugs than a triple-negative adenocarcinoma type, such as MDA-MD-231 (Mohapatra *et al*. 2014).

The mechanism of the cryptobrachytone C cytotoxic activity was not yet known. However, since the chemical structure of cryptobrachytone C was similar to other pyrone compounds, such as (R)-rugulactone isolated from *cryptocarya rugulosa* (Mohapatra *et al*. 2014), it was estimated that its cytotoxic activity would be similar to (R)-rugulactone. The difference between cryptobrachytone C and (R)-rugulactone was only in the C-2 group. In (R)-rugulactone, it had a vinyl group at C-2; whereas cryptobrachytone C had a hydroxyl group. With this similarity in structure, it was assumed that cryptobrachytone C had similarities with (R)-rugulactone activity. (R)-rugulactone was cytotoxic towards MCF-7 and MDA-MB-231 by the inhibition of the translocation of nuclear factor kappa B (NF-kB) (Mohapatra *et al*. 2014). NF-kB is a transcription factor that regulated gene expression, which played a role in the development and progression of cancer cells, such as cell proliferation and apoptosis (Duronio & Xiong 2013; Mohapatra *et al*. 2014). Based on the BrdU assay, cryptobrachytone C inhibited cell proliferation 12 h after treatment, which was thought to occur at the end of the G1 phase and at the beginning of the S phase of the doubling time of MCF-7 (Corbin *et al*. 2017; Bailon-Moscoso *et al*. 2017). Some natural substances that enter the cell could act as CDK inhibitors (Bailon-Moscoso *et al*. 2017). Cryptobrachytone C in MCF- 7 cells could act as a CDK inhibitor, where inhibition of CDK in G1 would affect the RB protein phosphorylation and the tumour suppressor p53. Inhibition of CDK would inhibit pRB phosphorylation from dissociating from E2Fs, so transcription of the E2Fs genes was inhibited and progression of the S phase was also inhibited (Duronio & Xiong 2013; Ruijtenberg & van den Heuvel 2016). Moreover, based on the results of the BrdU assay, which showed reduced DNA labelling by BrdU, they could indicate that the DNA synthesis was inhibited compared to the control. Inhibition of DNA synthesis or damage that occurred in the DNA triggered the tumour suppressor protein p53 to activate G1/S checkpoints so that the cell cycle would be stopped for repair, or apoptosis would be induced (Boik 2001; Bailon-Moscoso *et al*. 2017; Aubrey *et al*. 2018).

Activation of p53 by cryptobrachytone C induced apoptosis in the MCF-7 and T47D cells. Apoptosis of MCF-7 began to occur at cryptobrachytone C on IC_50_ 12.94 μM, although it was not significant when compared with the positive control of doxorubicin (Table 6). The apoptosis induction of the T47D cells was also not significantly different from the positive control (Table 7). In addition to the induction of apoptosis, cryptobrachytone C also induced necrosis in MCF-7 and T47D. The percentage of necrosis became larger along with the increasing concentration of cryptobrachytone C, in both the MCF-7 and T47D cells.

Apoptosis and cell necrosis caused a reduction in cell viability and proliferation, resulting in a low SF. This could be seen from the results of the clonogenic tests, which showed a decrease in the SF along with increased concentrations of cryptobrachytone C. The low SF was caused by the inhibition of the proliferation, so the number of cells in the colony decreased. The number of cells that could survive to form colonies was reduced, resulting in a reduced number of colonies formed. Reduced cell viability could also reduce the ability of cells to escape from the colony and migrate. This could be seen from the results of the migration tests, which showed the percentage of the inhibition of MCF-7 cell migration as 72.41 ± 9.27%, and 57.69 ± 4.16% in T47D.

Cryptobrachytone C as a new compound isolated from *Cryptocarya pulchrinervia*, an Indonesian indigenous plant, showed potential as an anti-cancer drug. Cryptobrachytone was cytotoxic, could inhibit proliferation, induced apoptosis, inhibited migration and inhibited the formation of colonies in MCF-7 cells. In T47D cells, cryptobrachytone C was not cytotoxic, but it induced proliferation, apoptosis, the inhibition of migration and colony formation.

## CONCLUSION

Cryptobrachytone C has antioxidant capabilities with an AAI index of 0.57, and it is categorised as a moderate antioxidant. It is cytotoxic to MCF-7 cell lines with IC_50_ 12.94 μM and in T47D cell lines with IC_50_ 65.32 μM. Cryptobrachytone C can inhibit proliferation, induce apoptosis, inhibit migration and inhibit the formation of the colony of MCF-7 and T47D cell lines.

## Figures and Tables

**Table 1:** Cytotoxic compounds of *Cryptocarya* spp. indigenous Indonesia.

Compound	Class	IC_50_ (μg/mL)	Species	References
Kamaharlactone		0.80	*C. kamahar*	(Juliawaty *et al*. 2020)
Goniothalamin		0.54		
Cryptopholione	Pyrone	2.94	*C. strictifolia*	(Juliawaty *et al*. 2020)
CP-1		3.04	*C. pulchrinervia*	(Juliawaty *et al*. 2020)
Cryptobrachytone C	3.03	0.23	*C. konishii*	
Infectocaryone				
Cryptocaryone	Calcon	0.01		
Desmethylinfectocaryone		0.62		(Kurniadewi *et al*. 2010)
Cryptocaryanone A	Flavanone	1.08	*C. cagayanensis*	
Cryptocaryanone B		0.13		
Laurolitsin	Alkaloid	5.20	C. arcboldiana	(Kurniadewi *et al*. 2010)

**Table 2:** Antiproliferative compounds from *Cryptocarya*.

Compound	Class	Species	Cell line	References
Cryptomoscatone D2		*C. mandioccana*	HeLa, SiHa, C33A	(GIocondo *et al*. 2009)
Cryptocaryone (CPC)	Pyrone	*C. concinna*	Ca9-22, CAL 27	(Chang *et al*. 2016)
Rugulactone		*C. rugulosa*	MCF-7, MDAMB-231	(Mohapatra *et al*. 2014)
Antofine and Dehydroantofine	Alkaloid	*C.chinensis*	L-1210, P-388, A-549, HCT-8	(Wu *et al*. 2012)

**Table 3:** Cryptobrachytone C cytotoxicities in MCF-7, T47D and MRC-5.

Cell lines	IC_25_ (μM)	IC_50_ (μM)	IC_75_ (μM)
MCF-7	4.31 ± 0.11	12.94 ± 0.32	38.89 ± 1.01
T47D	21.77 ± 0.78	65.33 ± 2.33	195.98 ± 6.99
MRC-5	40.89 ± 6.61	122.57 ± 19.84	368.05 ± 59.52

**Table 4:** Percentage of proliferation inhibition of MCF-7 cell lines induced by cryptobrachytone C.

Treatment	Cell proliferation (%)			Proliferation inhibition (%)		

	6 h	12 h	24 h	6 h	12 h	24 h
Cell control	100.00 ± 0.00	100.00 ± 0.00	100.00 ± 0.00	0.00 ± 0.00	0.00 ± 0.00	0.00 ± 0.00
Doxo 0.9 μM	12.09 ±1.77	7.95 ± 0.69	6.39 ± 4.46	87.91 ± 1.77	92.05 ± 0.69	93.61 ± 4.46
Cryptobrachytone C 4.31 μM	96.78 ± 0.23	77.41 ± 16.53	62.85 ± 14.22	3.22 ± 0.23	22.59 ± 16.53	37.15 ± 14.22
Cryptobrachytone C 12.94 μM	88.86 ± 7.34	70.67 ± 21.20	55.26 ± 9.71	11.14 ± 7.34	29.33 ± 21.20	44.74 ± 9.71
Cryptobrachytone C 38.89 μM	67.49 ± 22.78	46.06 ± 22.20	38.21 ± 17.20	32.51 ± 22.78	53.94 ± 22.20	61.79 ± 17.20

**Table 5:** Percentage of proliferation inhibition of T47D cell lines induced by cryptobrachytone C.

Treatment	Cell proliferation (%)			Proliferation inhibition (%)		

	6 h	12 h	24 h	6 h	12 h	24 h
Cell control	100.00 ± 0.00	100.00 ±0.00	100.00 ± 0.00	0.00 ± 0.00	0.00 ± 0.00	0.00 ± 0.00
Doxo 0.9 μM	5.81 ± 1.10	2.09 ± 1.39	1.62 ± 0.70	93.98 ± 1.10	97.63 ± 1.39	98.20 ± 0.79
Cryptobrachytone C 21.77 μM	72.98 ± 12.89	53.24 ± 8.81	40.67 ± 11.14	24.61 ± 12.89	45.04 ± 8.81	56.78 ± 11.14
Cryptobrachytone C 65.33 μM	62.59 ± 6.43	47.93 ± 3.31	35.81 ± 8.38	36.20 ± 6.43	51.42 ± 3.31	62.27 ± 8.39
Cryptobrachytone C 195.98 μM	41.09 ± 6.21	25.57 ± 2.79	18.04 ± 2.55	57.74 ± 6.21	73.89 ± 2.79	82.54 ± 2.54

**Table 6:** Apoptosis in MCF-7 induced by cryptobrachytone C.

Treatment	Average (%)			

	Living cell	Early apoptotic	Late apoptotic	Necrotic
Unstain	97.75 ± 0.02	1.14 ± 0.10	1.00 ± 0.04	0.11 ± 0.03
Doxo 0.9 μM	51.97 ± 0.61	16.91 ± 1.78	11.35 ± 0.02	19.78 ± 1.14
Cryptobrachytone C 4.31 μM	80.35 ± 0.78	12.68 ± 0.21	2.26 ± 0.18	4.88 ± 0.62
Cryptobrachytone C 12.94 μM	67.33 ± 5.37	14.75 ± 1.39	2.17 ± 0.09	15.75 ± 4.08
Cryptobrachytone C 38.89 μM	64.58 ± 2.99	15.02 ± 1.01	5.25 ± 0.40	15.15 ± 2.38

**Table 7:** Apoptosis in T47D induced by cryptobrachytone C.

Treatment	Average (%)			

	Living cell	Early apoptotic	Late apoptotic	Necrotic
Unstain	97.13 ± 0.52	1.75 ± 0.12	0.93 ± 0.39	0.30 ± 0.09
Doxo 0.9 μm	33.48 ± 5.73	55.58 ± 7.24	4.27 ± 3.02	6.67 ± 1.51
Cryptobrachytone C 21.77 μM	87.83 ± 1.13	7.12 ± 0.31	2.17 ± 0.24	2.88 ± 0.59
Cryptobrachytone C 65.33 μM	80.05 ± 2.57	14.17 ± 2.64	3.37 ± 0.66	2.42 ± 0.59
Cryptobrachytone C 195.98 μM	32.87 ± 7.68	52.15 ± 4.45	7.08 ± 1.81	7.90 ± 1.41

**Table 8:** Migration inhibition in MCF-7 and T47D cell lines induced by cryptobrachytone C.

MCF-7		T47D	

Treatment	Average (%)	Treatment	Average (%)
Cell control	0.00 ± 0.00	Cell control	0.00 ± 0.00
Doxo 0.9 μM	92.45 ± 4.46	Doxo 0.9 μM	82.75 ± 9.65
Cryptobrachytone C 4.31 μM	38.30 ± 9.92	Cryptobrachytone C 21.77 μM	34.28 ± 5.06
Cryptobrachytone C 12.94 μM	72.41 ± 9.27	Cryptobrachytone C 65.33 μM	57.69 ± 4.16
Cryptobrachytone C 38.89 μM	76.19 ± 4.73	Cryptobrachytone C 195.98 μM	62.47 ± 25.10

**Table 9:** Surviving fraction of MCF-7 and T47D cell lines.

MCF-7		

Treatment	Colony (∑)	SF
Cell control	16.50 ± 0.71	-
DMSO 0.1%	15.50 ± 2.12	0.47 ± -0.04
Cryptobrachytone C 4.31 μM	14.00 ± 1.41	0.43 ± 0.06
Cryptobrachytone C 12.94 μM	10.50 ± 0.71	0.32 ± 0.04
Cryptobrachytone C 38.89 μM	4.50 ± 0.71	0.14 ± 0.02

T47D		

Treatment	Colony (∑)	SF

Cell control	56.00 ± 9.90	-
DMSO 0.1%	41.50 ± 2.12	0.76 ± 0.17
Cryptobrachytone C 21.77 μM	36.50 ±4.95	0.85 ± 0.39
Cryptobrachytone C 65.33 μM	30.00± 1.41	0.75± 0.16
Cryptobrachytone C 195.98 μM	10.50 ± 3.54	0.20 ± 0.10
